# Evaluation of *Slug* expression is useful for predicting lymph node metastasis and survival in patients with gastric cancer

**DOI:** 10.1186/s12885-017-3668-8

**Published:** 2017-10-03

**Authors:** Han Hee Lee, Sung Hak Lee, Kyo Young Song, Sae Jung Na, Joo Hyun O, Jae Myung Park, Eun Sun Jung, Myung-Gyu Choi, Cho Hyun Park

**Affiliations:** 1 Department of Internal Medicine, The Catholic University of Korea, Seoul St. Mary’s Hospital, Seoul, Korea; 2 Department of Hospital Pathology, Seoul St. Mary’s Hospital, College of Medicine, The Catholic University of Korea, 222, Banpo-daero, Seocho-gu, Seoul, 06591 Republic of Korea; 3 Division of Gastrointestinal Surgery, Department of Surgery, Uijeongbu St. Mary’s Hospital, College of Medicine, The Catholic University of Korea, Seoul, Korea, 271, Cheonbo-ro Uijeongbu, Gyeonggi-do, 480-717 Republic of Korea; 4 Department of Radiology, The Catholic University of Korea, Uijeongbu St. Mary’s Hospital, Uijeongbu, Korea; 5 Department of Radiology, The Catholic University of Korea, Seoul St. Mary’s Hospital, Seoul, Korea; 6 Department of Surgery, The Catholic University of Korea, Seoul St. Mary’s Hospital, Seoul, Korea

**Keywords:** *Slug*, Gastric cancer, Epithelial–mesenchymal transition, Tissue microarray, Prognosis

## Abstract

**Background:**

*Slug* is a transcription factor that activates the epithelial–mesenchymal transition (EMT) process in cancer progression. The aim of our study was to evaluate the clinical significance of *Slug* expression in gastric cancer.

**Methods:**

The expression of *Slug* in gastric cancer tissues of 456 patients who underwent gastrectomy was evaluated by immunohistochemistry using tissue microarrays. *Slug* expression level was defined by the composite score determined by multiplying the tumor staining scores for intensity and extent. The associations of *Slug* expression with clinicopathological characteristics and overall and recurrence-free survival were analyzed.

**Results:**

Patients were divided into three groups according to *Slug* composite score (≤4, 6, and 9). Low, mid, and high expression of *Slug* was observed in 104 (22.7%), 130 (28.3%), and 225 (49.0%) of cases, respectively. Overall survival and recurrence-free survival progressively increased from high to low *Slug* expression. In terms of lymph node metastasis, the rate of positive lymph node metastasis was 38/104 (36.5%), 79/130 (60.8%), and 178/225 (79.1%) in low, mid, and high *Slug* expression groups, respectively, displaying a tendency to increase with higher *Slug* expression. In a multivariate analysis adjusting for patient age, tumor size, tumor depth, and histology, high *Slug* expression was associated with a high rate of positive lymph node metastasis compared with low *Slug* expression (odds ratio 3.42; 95% confidence interval, 1.74–6.69). In a subgroup analysis of T1 cancer, patients with negative *Slug* expression (defined as <5% positive tumor cells or no/weak staining) showed no lymph node metastasis (0/13), whereas those with positive *Slug* expression showed 15.9% (17/107) lymph node metastasis, with a negative predictive value of 100%.

**Conclusions:**

High expression of *Slug* in gastric cancer tissue was associated with lymph node metastasis and poor survival. Evaluation of *Slug* would be useful for discriminating patients at high risk of lymph node metastasis in early gastric cancer.

**Electronic supplementary material:**

The online version of this article (10.1186/s12885-017-3668-8) contains supplementary material, which is available to authorized users.

## Background

Gastric cancer is the third leading cause of cancer death worldwide, and almost 1 million new cases occur annually [1]. With the introduction of mass screening methods such as endoscopy and upper gastrointestinal series, the proportion of patients with early detection of early gastric cancer (EGC) or precancerous adenoma has been increasing [2, 3]. Endoscopic submucosal dissection (ESD) has become the standard therapy for EGC because it is minimally invasive and allows en bloc and complete resection [4]. Recently, there has been an attempt to expand the indications of ESD [5]. Along with this, prediction of lymph node metastasis (LNM) in EGC is becoming more important because LNM is one of the most important factors for assessment of prognosis and decision of therapeutic modalities [6, 7]. Advanced gastric cancer (AGC) has a particularly poor prognosis compared with EGC. AGC spreads locally by breaking through the gastric wall into neighboring tissue and metastasizes to regional lymph nodes. The presence of metastatic lymph nodes could be an outstanding prognostic factor. Differences in the prognoses of patients with negative lymph node metastasis versus positive lymph node metastasis are especially robust in surgically treated AGC [8–10].

Epithelial-mesenchymal transition (EMT) is a biologic process by which epithelial cells lose their cell-cell junctions and apical-basal polarity and gain a highly motile and invasive phenotype to become mesenchymal cells [11]. EMT is integral to embryo formation and organ development [12] and has also been shown to occur during wound healing and tissue fibrosis [13]. In cancer, EMT contributes pathologically to cancer progression by enabling primary tumor cells to break through the basal lamina and invade adjacent tissue, leading to tumor metastasis [14].

*Slug*, also known as Snail2, is one of the key transcription factors that activate EMT process in cancer progression [15]. It contributes to repression of the epithelial phonotype by binding to E-box DNA sequences in the proximal promoter region of the E-cadherin gene [16–18]. This role as a strong E-cadherin repressor mediates loss of tight junctions of epithelial cells and initiates EMT, which facilitates cancer cell invasion and distant metastasis [18, 19]. *Slug* has been highly studied in various cancers. In breast cancer patients, *Slug* is consistently overexpressed in aggressive and basal-type breast tumors [20] and seems to be involved in breast tumorigenesis and metastasis through regulation of the EMT [21]. It has also been demonstrated that *Slug* expression is correlated with poor prognosis in pancreatic and esophageal cancer patients [22, 23]. Recent studies have revealed that *Slug* not only functions in cancer metastasis, but also plays a role in cancer stemness [24, 25], implying that *Slug* participates in early steps of cancer progression.

In gastric cancer, upregulation of *Slug* mRNA is associated with suppression of E-cadherin in intestinal and diffuse type gastric carcinomas [26]. In a study focused on protein expression, high *Slug* expression was correlated with advanced stages and worse clinical outcomes [27]. However, there are only a few studies on the clinical significance of *Slug* in gastric cancer. In addition, the significance of *Slug* expression in early gastric cancer has not been proved.

Therefore, the purpose of our study was to evaluate the clinical significance of *Slug* expression in gastric cancer using a tissue microarray method in a large series of patients with resected gastric cancer.

## Methods

### Patients and clinical samples

A total of 459 patients (313 men and 146 women) were randomly selected by random number generation from 2495 consecutive patients with gastric cancer who had undergone radical surgery at Seoul St. Mary’s Hospital, The Catholic University of Korea, between 2000 and 2009. Clinicopathological data were reviewed retrospectively from the participants’ medical records and pathology reports at our institution. Variable factors including age, gender, type of surgery, tumor size, location, pathologic staging, histology, and lymphatic, venous, and perineural invasion were analyzed. Tumor location was categorized into upper, middle, and lower thirds of the stomach. The gastric cancers were staged according to the pathological tumor/node/metastasis (pTNM) classification (8th edition) of the Union for International Cancer Control [28]. The histological types of the gastric cancers were assessed according to the 2010 World Health Organization classification [29]. Tumors were also classified into intestinal, diffuse, and mixed types by Lauren classification [30]. Written informed consent was obtained from all patients. Patient consent and specimen collection were conducted in accordance with protocols approved by the Institutional Review Board of The Catholic University of Korea (KC14SISI0158).

### Tissue microarray construction and immunohistochemistry

All gastric specimens were histologically reviewed, and tissue microarrays (TMAs) were constructed from each of the formalin-fixed, paraffin-embedded (FFPE) tissue blocks using a Manual Tissue Arrayer (Beecher Instruments, Sun Prairie, WI, USA) with a 2.0-mm tip.

Immunohistochemical analysis was performed using primary antibody against Slug (ab188875) (polyclonal; 1:150; Abcam, Cambridge, UK). We determined the optimal dilution of the Slug antibody using positive control tissue such as normal gastric epithelial cells and placenta. Four-micrometer-thick tissue sections from the TMA blocks were transferred to Probe On Plus slides (Fisher Scientific, Pittsburgh, PA, USA) and baked for 2 h in a dry oven at 56 °C (Agilent Technologies, Santa Clara, CA, USA). The FFPE sections were deparaffinized in xylene three times and rehydrated through 100%, 90%, 80%, and 70% ethanol in Tris-buffered saline (pH 7.4). Antigen retrieval was achieved by boiling in 10 mM sodium citrate buffer (pH 6.0) using a microwave oven for 20 min. After treatment with 3% H_2_O_2_ in phosphate-buffered saline, the tissues were incubated with primary antibody at 4 °C overnight and then with diluted (1:100) biotinylated anti-mouse antibody (Abnova, Walnut, CA, USA) for 1 h at room temperature. The signal was amplified using diluted ExtrAvidin-peroxidase (1:50; Sigma-Aldrich, St. Louis, MO, USA) for 1 h at room temperature and visualized using the liquid 3,3′-diaminobenzidine + Substrate Chromogen system (Dako, Glostrup, Denmark). Counterstaining was performed with hematoxylin. Nonspecific staining was not observed in any negative control sections.

### Evaluation of immunohistochemical staining

Two pathologists (SH Lee and ES Jung) who were blinded to the clinicopathological parameters independently reviewed the immunohistochemical staining for the tissue sections. We used a semi-quantitative scoring system based on the intensity and extent of stained cells for each case. The staining intensity was graded from 0 to 3 (0 = no expression at all, 1 = weak, 2 = moderate, 3 = strong). The extent was graded from 0 to 3 (0 = <5%, 1 = 5–25%, 2 = 26–50%, 3 = >50%). The intensity scores and extent scores were multiplied to obtain the composite score.

### Statistical analysis

Continuous data are presented as mean ± standard deviation, and categorical data are presented as quantity and proportion. Pearson’s χ^2^ test for categorical variables and Student’s t test for unpaired data for continuous variables were performed to compare clinicopathological characteristics among the three *Slug* expression groups. A *P* value <0.05 was considered significant. Survival rates were calculated by the Kaplan–Meier method, with the date of gastrectomy as the starting point. Patients who were alive were censored at the time of the last follow-up. Differences in survival were examined by the log-rank test. Multivariable analysis was performed using a Cox proportional hazards model with a backward stepwise selection procedure. All analyses were performed by SAS for Windows software (version 8.02, SAS Institute, Cary, NC, USA).

## Results

### Expression profile of *Slug* in gastric cancer

**Fig. 1:**
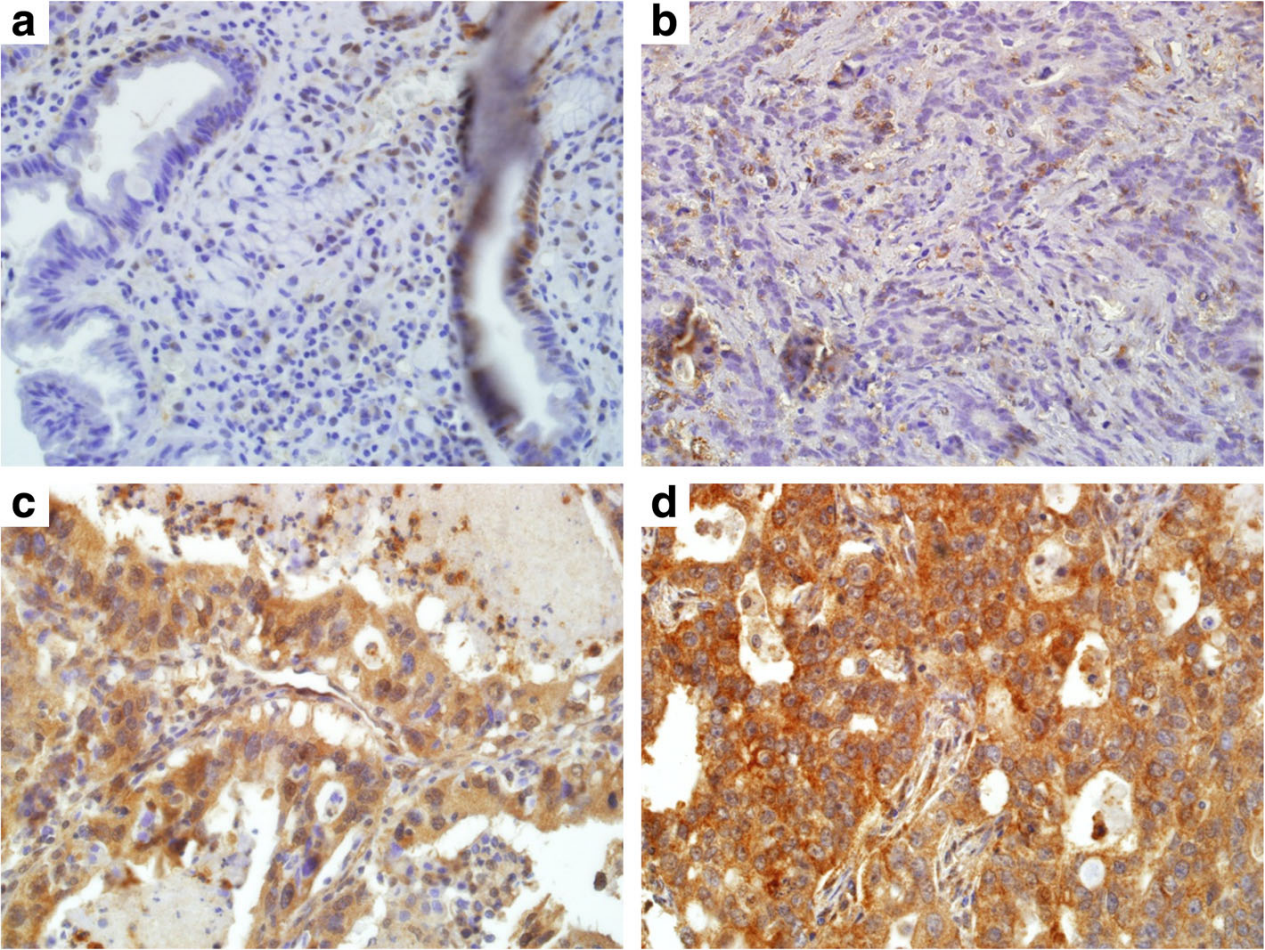
Immunohistochemistry findings showing expression of *Slug* in gastric cancer tissue. **a** no staining. **b** weak staining. **c** moderate staining. **d** strong staining

Table 1 shows overall immunohistochemical *Slug* expression in the gastric cancer tissue microarray. More than half of the tissues showed diffuse *Slug* expression, which corresponds to extent score 3, and 71.9% (330/459) of tissues showed intensity score 3, indicating strong staining. Figure 1 shows representative images of the range of *Slug* staining intensity. Multiplication of these two variables yielded the *Slug* composite score, which ranged from 0 to 9. Classification of the patients according to *Slug* composite score yielded 104 (22.7%), 130 (28.3%), and 225 (49.0%) patients in the low, mid, and high *Slug* groups, respectively.

**Table 1:** Scoring methods of *Slug* expression

Measures	Number	Percent
Extent		
0: negative (<5%)	15	3.3
1: sporadic (5–25%)	35	7.6
2: focal (25–50%)	141	30.7
3: diffuse (>50%)	268	58.4
Intensity		
0: no staining	2	0.4
1: weak staining	24	5.2
2: moderate staining	103	22.4
3: strong staining	330	71.9
Extent × Intensity		
= *Slug* composite score (range 0–9)		
Low (≤4)	104	22.7
Mid (6)	130	28.3
High (9)	225	49.0

### Relationships between *Slug* expression and clinicopathological parameters

Table 2 summarizes the clinicopathological characteristics of the 459 patients undergoing gastrectomy for gastric cancer. The mean age of the patients was 58.6 years (range 23–86 years), and 68.2% (*n* = 313) were male. Distal subtotal gastrectomy was the most commonly performed surgery (63.2%). The high *Slug* group tended to have large tumors and advanced tumor depth and stages. They also had a high rate of positive perineural invasion. Regarding histology, the proportion of poorly differentiated adenocarcinoma tended to increase from low to high *Slug* expression groups. However, the proportion of signet ring cell carcinoma was highest in the low *Slug* group.

**Table 2:** Comparison of characteristics of the patients according to *Slug* composite score

Measures	Total patients (*N* = 459)	Low (*n* = 104)	Mid (*n* = 130)	High (*n* = 225)	*P*
Age (years)					
Mean ± SD	58.6 ± 11.9	56.3 ± 12.3	58.9 ± 11.8	59.6 ± 11.6	0.064
Range	23–86	23–81	32–82	24–86	
Male	313 (68.2%)	65 (62.5%)	94 (72.3%)	154 (68.4%)	0.276
Type of surgery					
Total gastrectomy	166 (36.2%)	30 (28.8%)	51 (39.2%)	85 (37.8%)	
Subtotal gastrectomy	290 (63.2%)	73 (70.2%)	78 (60.0%)	139 (61.8%)	
Wedge resection	3 (0.7%)	1 (1.0%)	1 (0.8%)	1 (0.4%)	
Tumor size (cm)					
Mean ± SD	5.0 ± 2.9	4.1 ± 2.5	5.1 ± 3.3	5.4 ± 2.8	0.001
Range	0.2–19.0	0.4–12.5	0.5–19.0	0.2–15.5	
Location					
Upper third	80 (17.4%)	16 (15.4%)	22 (16.9%)	42 (18.7%)	0.599
Middle third	164 (35.7%)	43 (41.3%)	50 (38.5%)	71 (31.6%)	
Lower third	206 (44.9%)	44 (42.3%)	55 (42.3%)	107 (47.6%)	
Whole stomach	9 (2.0%)	1 (1.0%)	3 (2.3%)	5 (2.2%)	
Tumor depth (pT)					
T1	120 (26.1%)	56 (53.8%)	30 (23.1%)	34 (15.1%)	<0.001
T2	62 (13.5%)	18 (17.3%)	26 (20.0%)	18 (8.0%)	
T3	121 (26.4%)	14 (13.5%)	35 (26.9%)	72 (32.0%)	
T4	156 (34.0%)	16 (15.4%)	39 (30.0%)	101 (44.9%)	
TNM Stage					
I	131 (28.5%)	62 (59.6%)	37 (28.5%)	32 (14.2%)	<0.001
II	122 (26.6%)	25 (24.0%)	45 (34.6%)	52 (23.1%)	
III	206 (44.9%)	17 (16.3%)	48 (36.9%)	141 (62.7%)	
Venous invasion^a^					
Negative	406 (88.5%)	97 (94.2%)	117 (90.0%)	192 (85.3%)	0.055
Positive	52 (11.3%)	6 (5.8%)	13 (10.0%)	33 (14.7%)	
Perineural invasion					
Negative	270 (58.8%)	81 (77.9%)	75 (57.7%)	114 (50.7%)	<0.001
Positive	189 (41.2%)	23 (22.1%)	55 (42.3%)	111 (49.3%)	
Histology					
Adenocarcinoma					0.005^b^
Well differentiated	38 (8.3%)	12 (11.5%)	5 (3.8%)	21 (9.3%)	
Moderately differentiated	136 (29.6%)	23 (22.1%)	44 (33.8%)	69 (30.7%)	
Poorly differentiated	189 (41.2%)	35 (33.7%)	53 (40.8%)	101 (44.9%)	
Mucinous adenocarcinoma	19 (4.1%)	5 (4.8%)	7 (5.4%)	7 (3.1%)	
Signet ring cell carcinoma	77 (16.8%)	29 (27.9%)	21 (16.2%)	27 (12.0%)	
Lauren classification					
Intestinal	174 (37.9%)	40 (38.5%)	47 (36.2%)	87 (38.7%)	0.433
Diffuse	177 (38.6%)	40 (38.5%)	58 (44.6%)	79 (35.1%)	
Mixed	108 (23.5%)	24 (23.1%)	25 (19.2%)	59 (26.2%)	

Where appropriate, data are shown as the mean ± SD

^a^Lymphatic and venous invasion could not be evaluated in 2 and 1 cases, respectively

^b^Linear-by-linear association

### *Slug* expression and lymph node metastasis

The rate of positive lymph node metastasis was 36.5% in the low group, 60.8% in the mid group, and 79.1% in the high *Slug* expression group, thus displaying a tendency to increase with increasing *Slug* expression (Table 3). Positive lymph node ratio calculated by dividing number of metastatic LNs by number of retrieved LNs was significantly higher in the high *Slug* group. The high *Slug* group also showed a high proportion of positive lymphatic invasion.

**Table 3:** Association of lymphatic metastasis and *Slug* expression

Measures	Total patients (*N* = 459)	Low (*n* = 104)	Mid (*n* = 130)	High (*n* = 225)	*P*
Lymph node metastasis (pN)					
Negative	164 (35.7%)	66 (63.5%)	51 (39.2%)	47 (20.9%)	<0.001
Positive	295 (64.3%)	38 (36.5%)	79 (60.8%)	178 (79.1%)	
N1	98 (21.4%)	20 (19.2%)	30 (23.1%)	48 (21.3%)	
N2	99 (21.6%)	15 (14.4%)	26 (20.0%)	58 (25.8%)	
N3	98 (21.4%)	3 (2.9%)	23 (17.7%)	72 (32.0%)	
N3a	94 (20.5%)	3 (2.9%)	20 (15.4%)	71 (31.6%)	
N3b	4 (0.9%)	0 (0.0%)	3 (2.3%)	1 (0.4%)	
Number of metastatic lymph nodes	3.7 ± 4.9 (0–42)	1.2 ± 2.3 (0–12)	3.6 ± 5.8 (0–42)	4.9 ± 4.7 (0–25)	<0.001
Number of retrieved lymph nodes	42.4 ± 15.4 (6–106)	39.5 ± 13.3 (14–78)	44.9 ± 16.1* (8–97)	42.4 ± 15.8 (6–106)	0.028
Positive lymph node ratio	0.09 ± 0.12	0.03 ± 0.06	0.08 ± 0.12	0.12 ± 0.13	<0.001
Lymphatic invasion^a^					
Negative	154 (33.6%)	59 (57.3%)	45 (34.9%)	50 (22.2%)	<0.001
Positive	303 (66.0%)	44 (42.7%)	84 (65.1%)	175 (77.8%)	

^*^ *p* < 0.05; when compared with “low *Slug* composite score group” using the ANOVA test with post-hoc Tukey-HSD test

^a^Lymphatic invasion could not be evaluated in 2 cases

In a multivariate logistic regression analysis for lymph node metastasis, *Slug* composite score was identified as an independent predictive factor for lymph node metastasis even after adjusting for age, tumor size, tumor depth, and Lauren classification (Table 4). Compared with patients with low *Slug* score, the adjusted odds ratio in the high *Slug* group was 3.42 (95% confidence interval = 1.74–6.69). Tumor size and depth were also identified as predictive factors for lymph node metastasis.

**Table 4:** Multivariate analysis showing independence of the effect on lymph node metastasis

	Number of patients	Odds ratio	95% CI	*P*
Age		1.01	0.99–1.04	0.277
Tumor size		1.12	1.00–1.25	0.049
Tumor depth (pT)				
T1	120 (26.1%)	1 (ref)		
T2	62 (13.5%)	17.14	7.70–38.17	<0.001
T3	121 (26.4%)	33.99	14.87–77.71	<0.001
T4	156 (34.0%)	13.35	6.22–28.64	<0.001
Lauren classification				
Intestinal	174 (37.9%)	1 (ref)		
Diffuse + Mixed	285 (62.1%)	1.07	0.61–1.88	0.825
*Slug* composite score				
Low	104 (22.7%)	1 (ref)	1.09–1.76	
Mid	130 (28.3%)	1.33	0.67–2.63	0.413
High	225 (49.0%)	3.42	1.74–6.69	<0.001

**Fig. 2:**
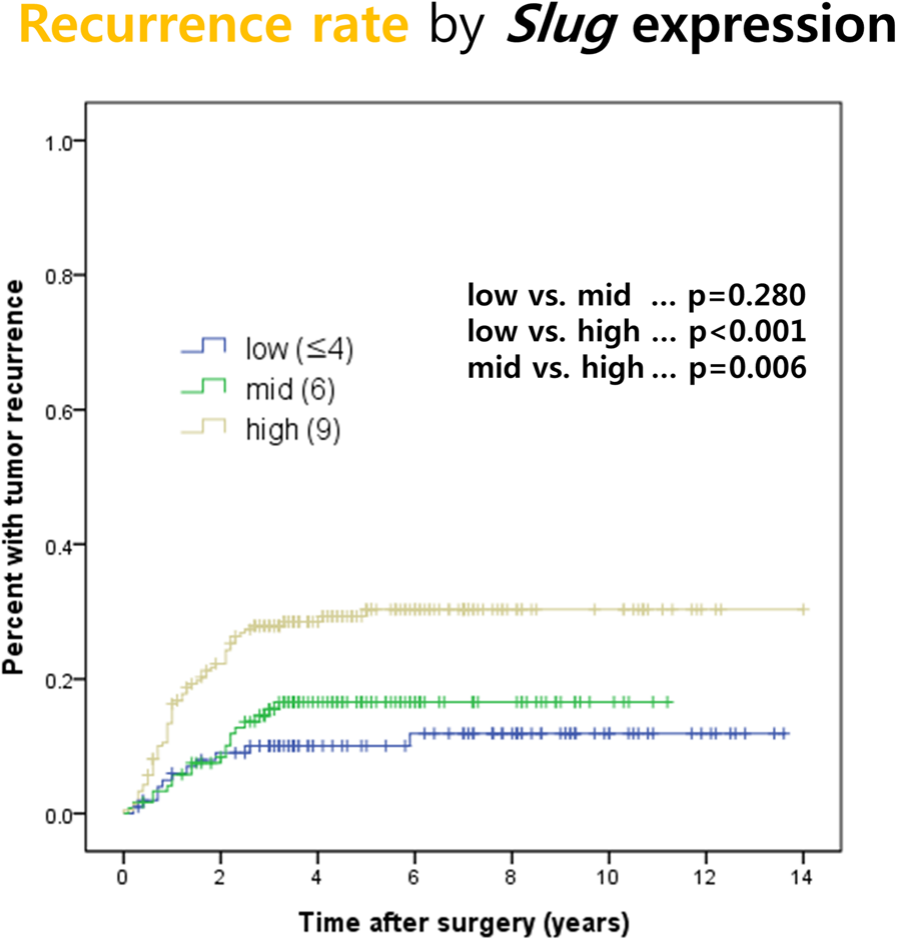
Cumulative recurrence rates according to *Slug* expression after gastrectomy

The recurrence rates of gastric cancer were compared between the three *Slug* groups (Fig. 2). Patients with high *Slug* score had the highest tumor recurrence rate. The rate of recurrence was significantly higher in the high *Slug* group than in the low (*P* < 0.001) and mid (*P* = 0.006) *Slug* groups. There was no statistically significant difference between the low and mid *Slug* groups (*P* = 0.280).

### *Slug* expression and survival

**Fig. 3:**
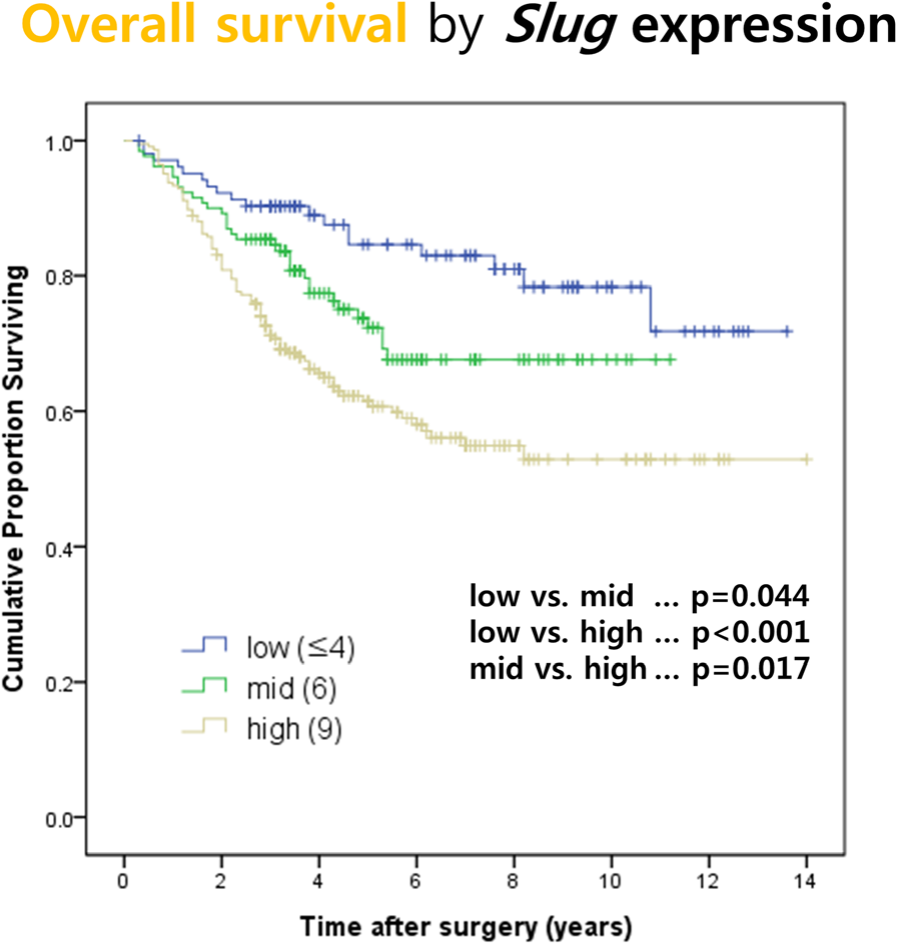
Overall survival according to *Slug* expression after gastrectomy

Overall survival rates were determined with respect to the *Slug* composite score using the log rank test (Fig. 3). The 5-year overall survival rate was significantly worse in the high *Slug* group compared with the mid (61.5% versus 72.4%; *P* = 0.017) and low (61.5% versus 84.6%; P < 0.001) *Slug* groups. The low *Slug* group had the best 5-year overall survival rate. In multivariable Cox regression analysis including age, gender, TNM stage, Lauren classification, and *Slug* composite score, *Slug* score was not significantly associated with overall survival, whereas age and TNM stage remained independent prognostic factors (Additional file 1: Table S1).

### Subgroup analysis of T1 tumors

We conducted a subgroup analysis of T1 tumors (Table 5). Negative *Slug* expression was defined as <5% positive tumor cells or no/weak staining intensity. Tumor depth and size were not significantly different between negative and positive *Slug* expression. Approximately 60% of cases with negative *Slug* expression were signet ring cell carcinoma.

**Table 5:** Subgroup analysis of T1 tumor according to *Slug* expression

	Total	*Slug* expression		*P*
		Negative (*n* = 13)	Positive (*n* = 107)	
Tumor depth				
T1a	56	6 (46.2%)	50 (46.7%)	0.969
T1b	64	7 (53.8%)	57 (53.5%)	
Tumor size	3.0 ± 1.9	2.7 ± 2.0	3.0 ± 1.9	0.670
Histology				
Adenocarcinoma, WD	25	0 (0.0%)	25 (23.4%)	0.001^a^
Adenocarcinoma, MD	37	3 (23.1%)	34 (31.8%)	
Adenocarcinoma, PD	31	2 (15.4%)	29 (27.1%)	
Signet ring cell cancer	27	8 (61.5%)	19 (17.8%)	
Lymph node metastasis				
Negative	103	13 (100%)	90 (84.1%)	0.210^b^
Positive	17	0 (0.0%)	17 (15.9%)	

*WD* well differentiated, *MD* moderately differentiated, *PD* poorly differentiated

^a^Linear-by-linear association

^b^Fisher’s exact test

The rate of lymph node metastasis in T1 tumor was 14.2% (17/120). Patients with negative *Slug* expression showed no lymph node metastasis (0/13), whereas those with positive *Slug* expression showed 15.9% (17/107) lymph node metastasis, with a negative predictive value of 100%.

## Discussion

The present study aimed to determine the relationship between *Slug* expression and prognosis in patients with gastric cancer. High *Slug* expression according to our composite score was observed in about 50% of gastric cancer tissues. We demonstrated that the expression of *Slug* is associated with tumor progression and poor prognosis in gastric cancer. Especially, *Slug* expression was highly correlated with various indicators reflecting lymphatic progression such as lymph node metastasis, lymphatic invasion, and positive lymph node ratio. As it is reasonable to consider that advanced cancer has greater migrating activity and invasiveness than EGC, this finding supports the hypothesis that *Slug*, one of the important EMT drivers, is involved in lymphatic metastasis of gastric cancer through the EMT process. In the case of T1 tumor confirmed after surgical resection, negative *Slug* expression might exclude lymph node metastasis of EGC.

To the best of our knowledge, there is only one previous study that investigated *Slug* protein expression in gastric cancer tissues by immunohistochemical staining [27]. In that study, 30% of tissues showed positive *Slug* expression, defined as detectable immunoreaction in the perinuclear and other cytoplasmic regions of more than 10% of the cancer cells. This is in contrast to findings from the current study showing that about 75% of gastric cancer patients had mid to high *Slug* expression. A possible explanation for this finding is that many more advanced cancers were included in our study compared to the previous study; approximately 60% of patients in the previous study were stage I, compared with only about 30% in our study. In another previous study based on mRNA expression of *Slug* by real-time quantitative RT-PCR, 58% of gastric cancer patients showed *Slug* upregulation in the tumor, which is in close agreement with our finding [26]. Moreover, the tendency for *Slug* expression to be associated with advanced pTNM stages was observed in both studies [26, 27]. The correlation of *Slug* expression with increased tumor size and perineural invasion was newly identified in the present study.

We focused on the association of lymphatic metastasis and *Slug* expression because *Slug* can activate the EMT process. For this purpose, we used the *Slug* composite score to produce a more continuous scale (low, mid, and high *Slug* groups) instead of dichotomizing the patient groups. As expected, higher *Slug* expression was associated with more prevalent lymph node metastasis and lymphatic invasion. In addition, the positive lymph node ratio gradually increased with increasing *Slug* score. This ratio represents lymph node metastasis density [31]. Much study has focused on this ratio because it has global prognostic relevance in gastric cancer regardless of stage in multivariable analysis and is more sophisticated than conventional nodal metastasis in TNM staging for predicting prognosis [32]. In addition, we demonstrated that *Slug* expression is an independent prognostic factor for lymph node metastasis in gastric cancer patients even after adjustment for well-known prognostic factors including tumor size and depth of tumor invasion.

The current study indicates that *Slug* expression correlates well with overall survival as well as tumor recurrence. The high *Slug* expression group had the worst long-term survival rate and the highest tumor recurrence rate. These results correspond well with previous studies, in which positive *Slug* expression was associated with distant metastasis and poor postoperative 5-year survival [26, 27]. To our knowledge, this is the first report of long-term survival and recurrence data according to *Slug* expression and suggests that EMT signaling with involvement of *Slug* could affect long-term prognosis after gastrectomy of gastric cancer patients.

In a subgroup analysis of T1 tumors, we documented that *Slug* expression is associated with unexpected lymph node metastasis in EGC. EGC is defined as gastric cancer that invades no more deeply than the submucosa, irrespective of lymph node metastasis [33]. It has been reported that about 10–15% of patients with EGC have lymph node metastasis [1, 34, 35]. Precise prediction of lymph node metastasis status in EGC is a very important issue because ESD has become increasingly popular as a minimally invasive treatment for EGC [36]. We applied strict criteria for negative *Slug* expression in order to increase the negative predictive value because false negative results could be fatal when making the decision between surgical resection and ESD. In our study, all patients with T1 tumor and negative *Slug* expression showed no lymph node metastasis even though some of them had submucosal tumor invasion (T1b) or undifferentiated (poorly differentiated or signet ring cell) type histology. Tumor depth beyond submucosa and histological differentiation are well known independent risk factors for lymph node metastasis of EGC [37, 38]. Interestingly, 8 of 13 Slug negative T1 tumors were signet ring cell cancer. A previous study showed that signet ring foci of 8 patients with hereditary diffuse gastric cancer had a low proliferative index and there was no evidence for EMT [39]. This finding corresponds well with our result.

Our study has some strengths. First, a relatively large number of patients were randomly selected from consecutive patients undergoing surgery for gastric cancer for TMA and analyzed. Moreover, we present a novel finding regarding greater than 5-year survival and tumor recurrence according to *Slug* expression. In addition, this is the first report to document the significance of Slug expression in EGC.

## Conclusions

Our data demonstrated that high expression of *Slug* in gastric cancer tissue was associated with higher tumor recurrence rate and poor long-term survival. In particular, in cases with lymph node metastasis *Slug* expression was an independent predictive factor regardless of tumor size or depth of tumor invasion. Negative *Slug* expression showed high negative predictive value for lymph node metastasis in EGC, which could have potential for future use in discriminating patients with EGC at high risk of lymph node metastasis.

## Additional file

Additional file 1: Table S1. Multivariate analysis showing independence of the effect on overall mortality. (DOCX 15 kb)

## Abbreviations

AGC: Advanced gastric cancer
EGC: Early gastric cancer
EMT: Epithelial–mesenchymal transition
ESD: Endoscopic submucosal dissection
FFPE: Formalin-fixed, paraffin-embedded
LNM: Lymph node metastasis
pTNM: Pathological tumor/node/metastasis
TMAs: Tissue microarrays
