# Supplementary material for: Evaluation of Slug expression is useful for predicting lymph node metastasis and survival in patients with gastric cancer
Source: BMC Cancer. 2017 Oct 3;17:670. doi: 10.1186/s12885-017-3668-8 (PMC5627408; doi:10.1186/s12885-017-3668-8)
Supplement: Additional file 1: Table S1. — Multivariate analysis showing independence of the effect on overall mortality. (DOCX 15 kb) [file 12885_2017_3668_MOESM1_ESM.docx]

| **Table S1.** Multivariate analysis showing independence of the effect on overall mortality | | | | |
| --- | --- | --- | --- | --- |
|  | Number of patients | Odds ratio | 95% CI | *P* |
| Age |  | 1.02 | 1.00–1.03 | 0.034 |
| Gender  Male  Female | 313 (68.2%)  146 (31.8%) | 1 (ref)  1.14 | 0.79-1.63 | 0.480 |
| TNM Stage |  |  |  |  |
| I | 131 (28.5%) | 1 (ref) |  |  |
| II | 122 (26.6%) | 3.97 | 1.80–8.75 | 0.001 |
| III | 206 (44.9%) | 11.54 | 5.62–23.72 | <0.001 |
| Lauren classification |  |  |  |  |
| Intestinal | 174 (37.9%) | 1 (ref) |  |  |
| Diffuse + Mixed | 285 (62.1%) | 1.08 | 0.75–1.56 | 0.670 |
| *Slug* composite score |  |  |  |  |
| Low | 104 (22.7%) | 1 (ref) |  |  |
| Mid | 130 (28.3%) | 0.96 | 0.53–1.73 | 0.889 |
| High | 225 (49.0%) | 1.07 | 0.63–1.84 | 0.796 |
|  | | | | |
